# Influence of Film Coating Thickness on Secondary Electron Emission Characteristics of Non-Evaporable Getter Ti-Hf-V-Zr Coated Open-Cell Copper Foam Substrates

**DOI:** 10.3390/ma15062185

**Published:** 2022-03-16

**Authors:** Jing Zhang, Jie Wang, Yong Gao, Yaocheng Hu, Yupeng Xie, Zhiming You, Sheng Wang

**Affiliations:** Shaanxi Engineering Research Center of Advanced Nuclear Energy, Shaanxi Key Laboratory of Advanced Nuclear Energy and Technology, School of Nuclear Science and Technology, School of Energy and Power Engineering, Xi’an Jiaotong University, Xi’an 710049, China; zhangjing1108@stu.xjtu.edu.cn (J.Z.); gaoyong1108@stu.xjtu.edu.cn (Y.G.); hyc1997@stu.xjtu.edu.cn (Y.H.); xieyupeng@stu.xjtu.edu.cn (Y.X.); youzm19960311@stu.xjtu.edu.cn (Z.Y.)

**Keywords:** accelerators, open-cell metal foams, film

## Abstract

The application of vacuum materials with low secondary electron yield (SEY) is one of the effective methods to mitigate the electron cloud (EC). In this study, the Ti-Hf-V-Zr non-evaporable getter (NEG) film was deposited on open-cell copper foams with different pore sizes for the suppression of electron multipacting effects. Besides, the influence of the film thickness on the secondary electron emission (SEE) characteristics of Ti-Hf-V-Zr NEG film-coated open-cell copper foam substrates was investigated for the first time. The results highlighted that all uncoated and NEG-coated foamed porous copper substrates achieved a low SEY (<1.2), which reduced at least 40% compared to the traditional copper plates, and the foamed porous coppers with 1.34-μm-thick NEG coating had the lowest SEY. Moreover, the surface chemistry and the morphological and structural properties of foamed porous coppers of different pore sizes with and without Ti-Hf-V-Zr NEG films were systematically analyzed.

## 1. Introduction

Low-energy electrons generated and accumulated in accelerators interact with circulating beam, leading to the formation of EC, accompanied with other detrimental effects, such as the increase in vacuum pressure [1], additional heat loads on the cryogenic vacuum system [2], and adverse impact on the stability of accelerated beam [3,4], etc. Beyond a certain electron density threshold, the EC can seriously affect the actual beam quality, and inducing beam instability and even disruption, as well as the degradation of the vacuum [5,6]. Those effects refer to the major limitations of present high energy colliders, such as the Large Hadron Collider (LHC) [7], the PEP-II collider [8], the CERN Super Proton Synchrotron (SPS) [9], Super KEKB [10,11], etc. Therefore, searching for reliable materials with low SEE to mitigate electron cloud effects (ECEs) is an essential technological objective.

The characteristics of high strength [12], a large surface area [13], excellent thermal properties [14], radiation features [15], and energy absorption capability [16] make Open-cell Metal Foams (OCMFs) an attractive material to be applied for radiation shielding and beam liner design [17,18] in particle accelerators. It is known that the geometrical modifications on a material surface can effectively suppress SEE from it [19,20,21,22]. Multiple reflections may take place when an electron hits a rough surface, which correspondingly enhances the probability of capturing electrons. Meanwhile, porous metal foams have the potential to create a rougher surface with a larger area and a higher depth-to-spacing ratio than current smooth-surface substrates [23]. R. Cimino et al. [24] proposed and studied some characteristics of foamed porous coppers with a pore size greater than 500 µm, especially their qualification in terms of SEY. Moreover, some characteristics of foamed porous coppers for beam screen were demonstrated as well, such as excellent vacuum behavior, low surface resistance, good mechanical structural properties, etc. [17,24]. These benefits suggested some potential use of the foamed porous coppers as an EC moderator in the accelerator technology. The effect of foamed porous coppers with a pore size less than 500 µm on the SEY property was studied in this article.

It is known that the enhancement and sustainability of the vacuum is important for particle accelerators, which can be effectively solved by coating the non-evaporable getters (NEGs) with micron/sub-micron thickness on the inner wall of the vacuum pipe [25,26,27]. The research by Malyshev O.B. et al. [28,29] demonstrated that the Ti-Hf-V-Zr NEG film with a columnar thin film structure showed a good pumping capacity and a low electron-stimulated desorption (ESD) yield after heated at the activation temperature of 140–150 °C for 24 h. In addition, the NEG film could not only adsorb residual gas in the vacuum chamber but also provide a low secondary electron yield [30]. In this study, the Ti-Hf-V-Zr NEG film and the open-cell copper foam were proposed to intrinsically produce low-SEY surfaces and simultaneously maintain vacuum stability in the accelerator. The macroscopically grooved surfaces of foamed porous coppers coated with Ti-Hf-V-Zr films were proposed and studied to reduce the SEY and hence mitigate ECEs. Besides, the Ti-Hf-V-Zr coatings deposited on foamed porous copper substrates with a large surface area also provided a good pumping capacity in the beam vacuum system.

Here, the SEY property of porous copper substrates with different pore sizes less than 500 μm and the influence of the coverage of different NEG equivalent thicknesses on the SEY of foamed porous coppers were discussed for the first time. The trends of the SEY (*δ*) corresponding to the changes in film thickness were quantified to reveal the differences among the SEYs of Ti-Hf-V-Zr NEG coatings with different NEG equivalent thicknesses. Thus, the Ti-Hf-V-Zr NEG films were deposited on foamed porous copper substrates with different pore sizes and in each case with a range of thicknesses. Besides, the surface morphology, cross-sectional morphology, surface microstructure, and chemistries of foamed porous coppers before and after NEG coating were analyzed.

## 2. Materials and Methods

### 2.1. Sample Preparation and Cleaning Procedures

Foamed porous coppers were purchased from Longshengbao Electronic Materials Co., Ltd. (Kunshan, China), with the average pore diameters of ~100, ~300, and ~500 µm produced by the electron deposition process. The polished Si wafers were purchased from Topvendor Technology Company (Beijing, China) to measure the film thickness. Before deposition, all samples were ultrasonically cleaned in acetone and ethyl alcohol solutions for 10 min, respectively, to remove the impurities.

### 2.2. Film-Coating Equipment

For the purpose of evaluating the effect of different Ti-Hf-V-Zr film thicknesses of coated and foamed porous coppers with different pore sizes on SEY, the Ti-Hf-V-Zr films were deposited on Si<111> single-crystal and foamed porous copper substrates by the direct current (DC) magnetron sputtering (Chuangshiweina, Beijing, China) method. Si substrates were used to evaluate the sputtering rate taken as a reference, and the thickness of the films was controlled by the duration of deposition. An alloy target consisting of Hf, Ti, Zr, and V elements with an atomic ratio of 1:1:1:1 was used. The deposition was carried out with the background pressure of ~5.8 × 10^−4^ Pa, the working gas pressure of 0.5 Pa, the Ar gas flow of ~20 sccm, and the discharge power of 280 W, as well as the cathode voltage of ~371 V, the sputtering current of ~0.76 A, and the distance from samples to the target of 8 cm. Due to the unevenness of the porous copper surface, the “NEG equivalent thickness” in this study was used to represent the “film thickness.” During the Ti-Hf-V-Zr NEG film deposition, the temperature of the sample disc increased from 19 °C to 71 °C. The target was pre-sputtered for 3 min to remove impurities on its surface, while the substrates were blocked by the movable disks. The information of the samples are shown in Table 1.

### 2.3. Characterization Method

The surface and cross-sectional morphologies of foamed porous copper samples before and after the Ti-Hf-V-Zr NEG film deposition were measured by a JEOL 7800F Schottky field scanning electron microscope (SEM, Japan Electron Optics Laboratory, Tokyo, Japan) and a X-ray diffractometer (XRD, SHIMADZU, Kyoto, Japan) with a copper Kα radiation (λ = 0.154 nm). The 2θ data were collected between 30° and 80° at a speed of 2°/min. The cross-sectional elemental compositions of the NEG film coatings were characterized by using a Zeiss GeminiSEM 500 emission scanning electron microscope (FE-SEM, Carl Zeiss GmbH, Hallbergmoos, Germany) with an energy dispersive spectrometer (EDS) system. The surface chemical state was analyzed by using AXIS ULtrabld X-ray Photoelectron Spectroscopy (XPS, Kratos, Manchester, UK) at a working pressure of ~10^−7^ Pa. An individual sample with an area of 10 × 10 mm^2^ was mounted on the sample holder. All XPS data were obtained with a 150 W Al Ka X-rays operator and a 45° analyzer at the beam size of 300 × 700 μm^2^. The test area of the sample was 2 × 2 mm^2^. Here, the XPS results were analyzed by CasaXps (2.3.17PR1.1, 2015, Casa Software Ltd, Devon, UK). C 1s peak with the binding energy (BE) of 284.8 eV was used for the binding energy calibration. Based on the multiple sets of data tested, the error of XPS chemical composition results was about ±0.3 at%. The SEY measurement was carried out with a primary electron dose of 7 × 10^−6^ C∙mm^−2^ with a current of 8 nA. During the SEY test, the base pressure in the test chamber was below 2 × 10^−7^ Pa and the beam spot with a diameter of 1 mm. The test error of SEY was within ±5%. The SEY measuring facility is introduced in detail in Ref. [31].

## 3. Results and Discussion

### 3.1. Surface and Cross-Section Morphology

**Figure 1:**
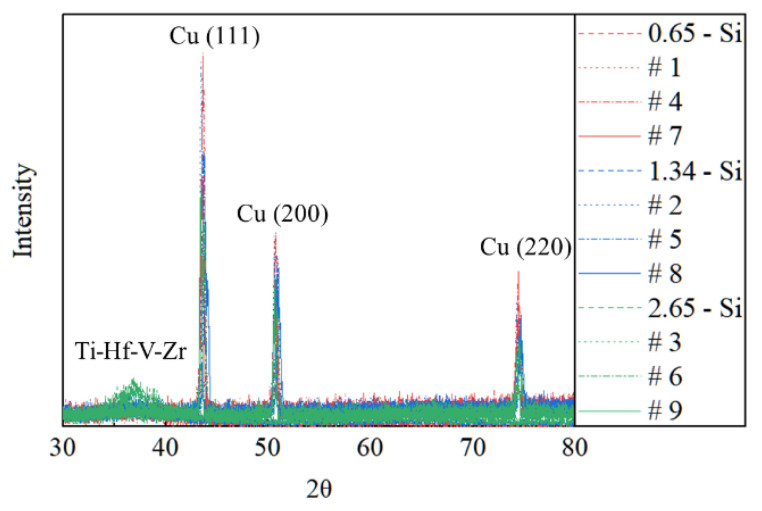
XRD of Ti-Hf-V-Zr films deposited on silicon and foamed porous copper substrates: 0.65 µm, 1.34 µm, and 2.65 µm thick NEG-coated Si substrates; samples #1, #2, #3, #4, #5, #6, #7, #8, and #9.

SEM and XRD were utilized to study the structures and morphologies of all the samples. The structures of the Ti-Hf-V-Zr NEG films were investigated by XRD for the Si and foamed porous copper substrates, as shown in Figure 1. The peaks for the NEG films were similar in both cases. The spectrum indicates that Ti, Zr, and V were hexagonal close-packed (hcp) structures, and Hf had a body-centered cubic (bcc) structure. The peaks at 2θ = 43.29°, 50.43°, and 74.13° in the XRD pattern were ascribed to (111), (200), and (220) of copper, respectively (the same as seen previously in reports [32,33,34]). The 2θ position of the major XRD peaks is in the 2θ range of 30–40° [28,29,35]. Here, the broad peaks associated with the Ti-Hf-V-Zr film occur at 2θ = 36.5° and 2θ = 37.1° for Si and foamed porous copper substrates, respectively. Calculated by the Scherrer equation, the average grain sizes of the Ti-Hf-V-Zr coatings deposited on Si and foamed porous copper substrates were 1.40 nm and 1.32 nm, respectively. This means that the surface conditions have a negligible effect on the crystallite size and structure of the Ti-Hf-V-Zr coatings.

**Figure 2:**
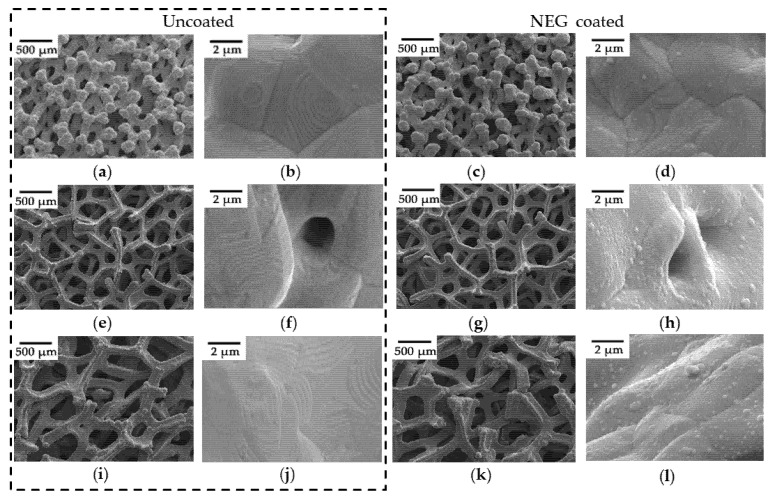
The SEM micrographs of the uncoated/NEG coated porous copper with average pore sizes of 100, 300, and 500 µm, respectively. (**a**,**b**) Uncoated, 100 µm; (**c**,**d**) 0.65-µm-thick NEG coated, 100 µm. (**e**,**f**) Uncoated, 300 µm; (**g**,**h**) 0.65-µm-thick NEG coated, 300 µm. (**i**,**j**) Uncoated, 500 µm; (**k**,**l**) 0.65-µm-thick NEG coated, 500 µm.

The morphologies of the as-prepared cleaned foamed porous coppers with and without coating were obtained by SEM (Figure 2). It can be revealed from Figure 2a,e,i that the foamed porous coppers are composed of connective pores (like a sponge) and a three-dimensional (3D) reticulated structure. The wave-like sintered texture on the surface of the foamed porous coppers can be seen with higher resolution. Besides the macropores, spherical micropores with a diameter less than 3 µm (Figure 2f) can also be observed on the surface of the foamed porous copper.

**Figure 3:**
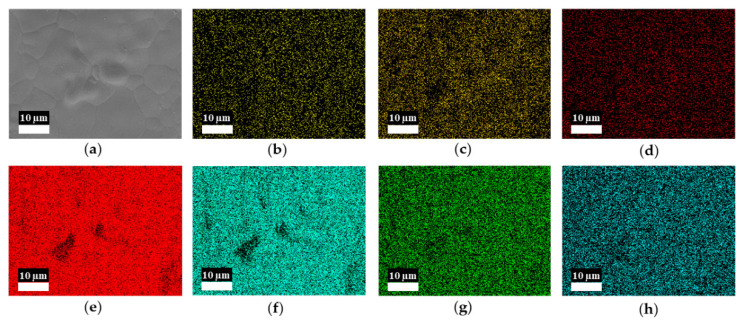
EDS mappings of the 0.65-µm-thick Ti-Hf-V-Zr NEG-film-coated 500 μm pore size Cu foams. Original (**a**) and corresponding Cu (**b**), C (**c**), O (**d**), Hf (**e**), Zr (**f**), Ti (**g**), and V (**h**).

After depositing a 0.65-µm-thick Ti-Hf-V-Zr layer (Figure 2c,d,g,h,k,l), the 3D structure remained almost the same but featured a rougher surface. The EDS mappings (Figure 3a–h) show the elemental analysis of the 0.65-μm-thick Ti-Hf-V-Zr NEG film coated on foamed porous copper substrates. There are some dark areas on the surface, and Ti, Hf, V, Zr, C, O, and Cu elements can be seen as well. This may be caused by uneven grooves on the sample surface and the accuracy of the EDS instrument [36]. Overall, the EDS results suggested that the Ti-Hf-V-Zr NEG coating was successfully deposited on the surface.

**Figure 4:**
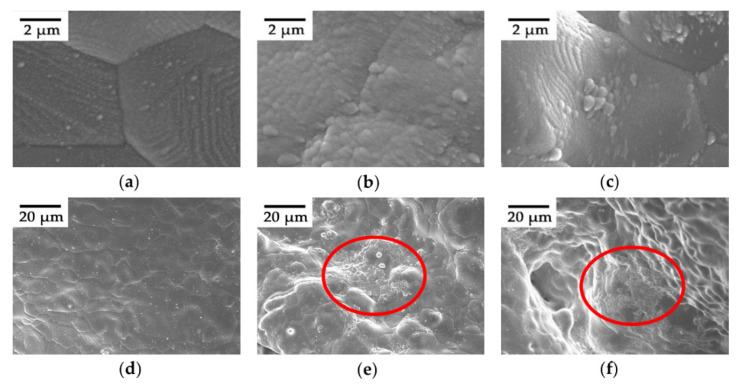
SEM micrographs of different areas on the surface of sample #9. (**a**−**c**) film surface morphologies with a scale of 2 µm and (**d**−**f**) with a scale of 20 µm.

Figure 4 shows the SEM images of different areas on the surface of the foamed porous copper with a pore size of 500 µm and a 2.65-µm-thick NEG film (Sample #9), which facilitates the exploration of the deposition behavior of NEG on foamed porous coppers. Meanwhile, it can be seen from this figure that the NEG film has an island-like structure with a size ranging from nm to µm (Figure 4a–c). As shown in Figure 4c, it is deposited on the scaffold in the form of island-like aggregation, but in sharp contrast, it is densely deposited on the scaffold of the foamed porous copper (Figure 4a,b). Figure 4d–f show that within the same deposition time, the film density on the local surface of the sample varies greatly (emphasized by red circles in Figure 4e,f), which is affected by the self-shadowing effect caused by the 3D structure of open-cell metal foams. It is worth mentioning that the growth morphology of the film of varied thicknesses on different substrates is the same according to our SEM experimental results.

**Figure 5:**
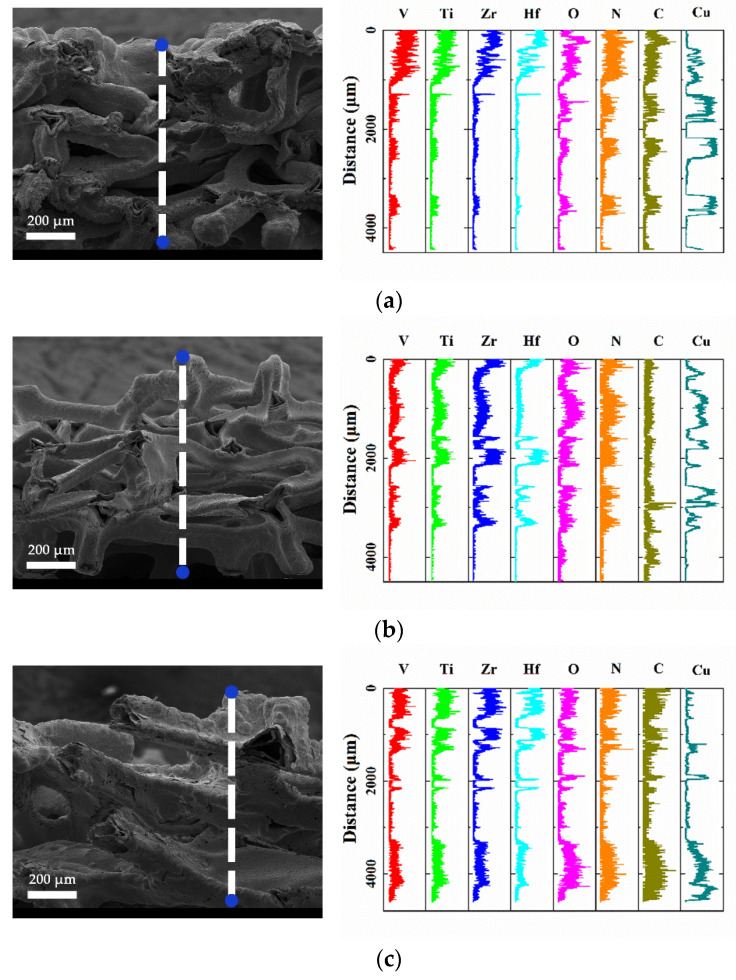
Cross-sectional SEM images and EDS line scans of 1.34-µm-thick Ti-Hf-V-Zr NEG-coated open-cell copper foam substrates with an average pore size of 100, 300, and 500 µm: (**a**): 100 µm, sample #2; (**b**) 300 µm, sample #5; (**c**) 500 µm, sample #8.

The samples were brittlely fractured at room temperature (RT) to produce the fresh cross-section surface after immersion in the 77 K liquid nitrogen for ~5 min. The cross-sectional SEM micrographs and the EDS line scannings of Samples #2, #5, and #8 are shown in Figure 5. The EDS line scanning results showed that the deposition of the coating mainly occurred at the outer surface, because the Cu element was barely found on it but the partial coating on the inner surface. Additionally, the contents of Ti, V, Hf, and Zr elements obviously decreased from the outer surface of the substrate to the inner surface. Compared with Samples #5 and #8, it is noteworthy that the line scanning of Sample #2 showed a low content of Ti, V, Hf, and Zr elements on the substrates, and only weak Ti, V, Hf, and Zr signals were observed on the inner pore substrate surface. For open-cell porous materials, the outer surface area is much smaller than the inner porous surface area [16]. It can be seen from the micrographs in Figure 5 that the film covers the inner surface of the 3D structure of the porous copper, suggesting that the 3D structure is beneficial for the increase in surface area in film growth. Besides, Figure 5 also reveals that this foam metal skeleton has a hollow throughhole structure.

### 3.2. Surface Composition

**Figure 6:**
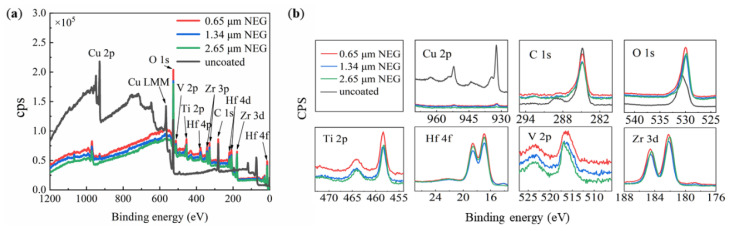
The XPS spectra of (**a**) uncoated (black) open-cell copper foam with pore size of 300 µm, Ti-Hf-V-Zr NEG film coated open-cell copper foam (Sample #4, red), (Sample #5, blue), and (Sample #6, Green); (**b**) the XPS survey scan of Cu, C, O, Ti, Hf, V, and Zr in the Ti-Zr-V-Hf film with foamed porous copper substrates.

It was reported that the further increase in the SEY may result in the introduction of impurities such as carbon and oxygen on the sample surface [37,38]. As an example, the states of the XPS-determined surface of NEG-coated and uncoated foamed porous coppers with a pore size of 300 μm were characterized. As shown in Figure 6b, it can be seen that after coating, the shake-up of the peaks corresponding to Cu(II) almost disappeared at 933 eV. In detail, the relative contents of Cu, C, O, Ti, V, Hf, and Zr in each state of Samples #4–6 were compared by XPS calculations, as shown in Table 2. The XPS survey spectra indicate that the ratios of Cu, Ti, V, Hf, and Zr elements of Sample #4 (with a film thickness of 0.65 μm), Sample #5 (film thickness of 1.34 μm), and Sample #6 (with a film thickness of 2.65 μm) were around 0.35 at%, 4.83 at%, 1.79 at%, 1.01 at%, 5.31 at%, 0.26 at%, 4.75 at%, 1.93 at%, 1.43 at%, 5.09 at%, 0.09 at%, 5.32 at%, 2.35 at%, 1.64 at%, and 5.78 at%, respectively. Besides, the XPS results revealed that carbon and oxygen contaminations could be found on the NEG film surfaces. The percentage of C significantly decreased about 29.25–38.6% after coating, while that of O increased about 24.7–33.8%. Moreover, the further oxidation of Ti-Hf-V-Zr during the sample transfer process led to a further increase in the O element. The occurrence of these metal oxides is consistent with Ti 2p, Hf 4f, V 2p, and Zr 3d spectra, and the signals of titanium oxide (Ti^4+^; BE at 458.7 eV), hafnium oxide (Hf^4+^; BE at 17.0 eV and 18.0 eV), zirconium oxide (Zr^4+^; BE at 182.3 eV and 184.7 eV), and vanadium oxide (V^3+^; BE at 517.5 eV) were observed.

### 3.3. SEY Results

**Figure 7:**
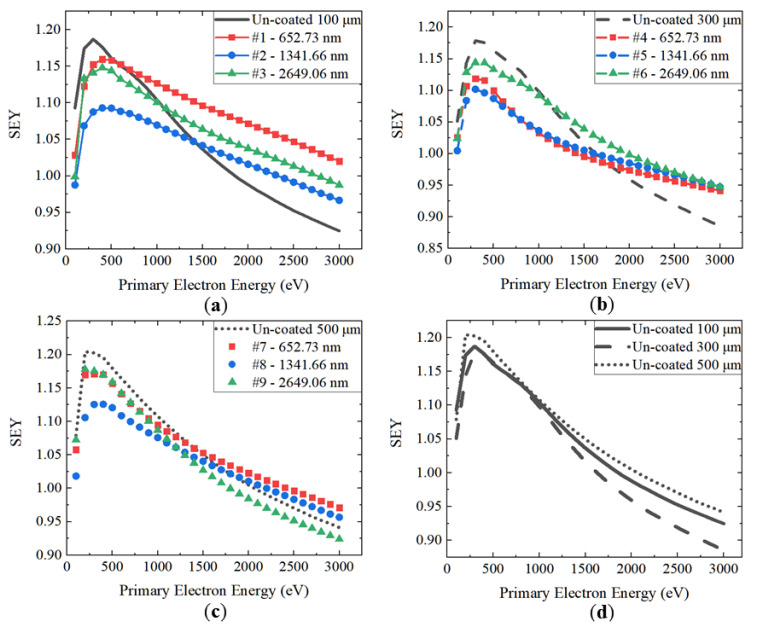
The SEY curves of foamed porous copper substrates with and without coating. (**a**) Uncoated foamed porous copper with 100, 300, and 500 μm pore size. (**b**) Samples #1, #2, and #3 and uncoated 100 μm. (**c**) Samples #4, #5, and #6 and uncoated 300 μm pore size. (**d**) Samples #7, #8, #9, and uncoated 500 μm.

The SEY properties of foamed porous coppers before and after NEG coating are summarized in Table 3, and the dependence of the maximum SEYs (*δ_max_*) as a function of incident electron energy is shown in Figure 7a–d. As shown in Table 3, the primary electron energy (*E_p_*) varied from 100 to 3000 eV, and the *δ_max_* of uncoated foamed porous copper of 100, 300, and 500 μm were 1.19, 1.18, and 1.20, respectively. Compared with *δ_max_* ≈ 2.0 at *E_p_* ≈ 300 eV, which was found for flat coppers [39,40,41,42], the *δ_max_* of the foamed porous copper substrates decreased about 40%.

**Figure 8:**
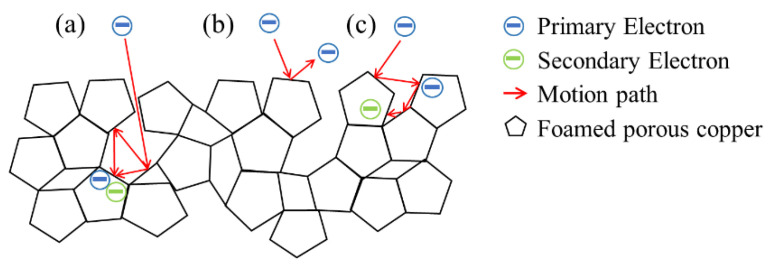
Schematic example of SEE from foamed porous copper. (**a**) Primary electron impact hole trap structure. (**b**) Primary electron impact top of the surface. (**c**) Primary electron impact uneven 3D surface.

Secondary electrons are induced when primary electrons impact on the surface of the material. As shown in Figure 8, when they are induced into the uneven 3D surface of the foamed porous copper, the pores and uneven grooves trap secondary electrons (Figure 8a,c), and they only partially escape to the outside (Figure 8b), while the rest are trapped in the network-like open pore structure. Therefore, the probability that the electrons can escape from the open-cell copper foam surface is reduced, resulting in a lower SEY.

SEY thresholds have thus been evaluated for Large Hadron Collider arcs with a *δ_max_* less than 1.5 [43]. Beyond this value, the beam-induced multipacting occurs. However, to suppress the build-up of EC, a surface treatment with a SEY no greater than 1.0 may be required in the Future Circular Collider: The Hadron Collider (FCC-hh) [44]. There is no doubt that open-cell copper foam possesses unique advantages in terms of SEY reduction as a simple and economical material. However, the SEY changing trend of the uncoated foamed porous copper substrates with pore sizes ranging from 100 to 500 μm was unobvious.

An earlier study [45] showed that the *δ_max_* of the 2.2-µm-thick quaternary Ti-Hf-V-Zr NEG-film-coated flat copper substrates was 1.37 at *E_p_* ≈ 300 eV and that the *δ_max_* of the foamed porous coppers covered by a 2.65-µm Ti-Hf-V-Zr film (Sample #3, #6 and #9) decreased about 0.19–0.22, compared to that of the NEG-coated flat coppers. Moreover, the NEG-coated foamed porous copper substrates have SEYs of 2.5–5.1%, 5.8–8.4%, and 1.7–3.4% after being coated with the Ti-Hf-V-Zr NEG film with a thickness of 0.65, 1.34, and 2.65 μm, which is lower than those of the uncoated foamed porous copper substrates. This indicates that foamed porous coppers with a 1.34-μm-thick Ti-Hf-V-Zr NEG coating have the lowest SEY, compared to others with different thicknesses investigated in this study. It was also found that the 1.34-μm-thick Ti-Hf-V-Zr NEG coating reduces the *δ_max_* of the 300 μm foamed porous coppers from 1.19, 1.18, and 1.20 to 1.09, 1.10, and 1.13, respectively. 

As shown above, the foamed porous copper coated with the Ti-Hf-V-Zr NEG film is a promising method of reducing SEY to below 1.2 for electron cloud mitigation, which can be considered to be applied in the accelerator vacuum systems.

## 4. Conclusions 

Open-cell copper foams before and after the Ti-Hf-V-Zr NEG coating were proposed to mitigate the EC in accelerators and other vacuum devices. The NEG film was deposited on the foamed porous copper substrates of three different pore sizes and in each case with three different thicknesses. For the first time, the relationship between the quaternary NEG film thickness on foamed porous copper samples and the SEY was systematically investigated. The results of this study are summarized as follows:

(1) The 3D reticulated structure on the foamed porous copper surface with a pore size of 100–500 μm can significantly reduce the SEY. It is clear that the network structure in foam geometries can reduce the SEY since the uneven 3D regular surfaces and pores trap the secondary electrons. Compared with flat coppers, the SEY of the open-cell copper foam can be reduced by at least 40%, and the foamed porous copper has a promising application potential for reducing SEY to below 1.2. However, the *δ_max_* of foamed porous coppers with a pore size less than 500 μm decreases insignificantly.
(2) The *δ_max_* of the NEG-coated foamed porous copper substrates is lower than that of the uncoated ones. After coated with the Ti-Hf-V-Zr NEG film with a NEG equivalent thickness of 0.65, 1.34, and 2.65 μm, the foamed porous coppers had the maximum SEYs that were reduced by 2.5–5.1%, 5.8–8.4%, and 1.7–3.4%, respectively. However, when coated with a 1.34-μm-thick NEG film, such foamed porous coppers had the lowest SEY.
(3) The Ti-Hf-V-Zr films have nanocrystalline structures on both Si and foamed porous copper substrates. XPS results show that the NEG film containing impurities such as carbon and oxygen were partially oxidized. Besides, the Ti-Hf-V-Zr NEG film was unevenly deposited on the foamed copper substrate. The EDS line scanning maps indicate that the inner surface of foamed porous coppers with high depth-to-spacing ratios was also covered by a quaternary NEG film, which was thus provided with a large specific surface area. Moreover, the combination of the NEG film with foamed porous copper substrates could further reduce the SEY. Therefore, the NEG coated open-cell copper foam walls seem to be a promising method to solve problems of high vacuum and EC in the future particle accelerators.

## Figures and Tables

**Table 1:** Detailed parameters for NEG coating deposition.

Sample	#1	#4	#7	#2	#5	#8	#3	#6	#9
**Pore size/µm**	100	300	500	100	300	500	100	300	500
**Deposition Time/s**	448			896			1792		
**NEG equivalent thickness/µm**	0.65 ± 0.017			1.34 ± 0.055			2.65 ± 0.135		

**Table 2:** XPS (at%) analysis for quantification of the concentrations of the copper foams with pore size of 300 µm before and after coating.

Sample	Cu	C	O	Ti	V	Hf	Zr
uncoated	6.68	55.29	38.03	0	0	0	0
#4	0.35	38.16	48.56	4.83	1.79	1.01	5.31
#5	0.26	39.12	47.41	4.75	1.93	1.43	5.09
#6	0.09	33.94	50.88	5.32	2.35	1.64	5.78

**Table 3:** The SEY properties of samples used in this study.

Sample	Pore Size/μm	NEG Thickness/μm	*δ_max_*	*E_max_*/eV
uncoated	100	0	1.19	300
#1		0.65	1.16	400
#2		1.34	1.09	400
#3		2.65	1.15	400
uncoated	300	0	1.18	300
#4		0.65	1.12	300
#5		1.34	1.10	300
#6		2.65	1.14	300
uncoated	500	0	1.20	300
#7		0.65	1.17	400
#8		1.34	1.13	300
#9		2.65	1.18	300

## Data Availability

Not applicable.
